# Pegylated interferon may be considered in chronic viral hepatitis E resistant to ribavirin in kidney transplant recipients

**DOI:** 10.1186/s12879-020-05212-2

**Published:** 2020-07-16

**Authors:** I. Ollivier-Hourmand, L. Lebedel, A. Lecouf, M. Allaire, T. T. N. Nguyen, C. Lier, T. Dao

**Affiliations:** 1 Department of Gastroenterology, University Hospital, Avenue de la côte de Nacre, 14000 Caen, France; 2 Department of Nephrology, University Hospital, Caen, France; 3 Inserm U1149, Center for Research on Inflammation, Faculté de Médecine Xavier Bichat, Université Paris Diderot, Sorbonne Paris Cité, Paris, France; 4 Department of Virology, University Hospital, Caen, France

**Keywords:** Hepatitis E, Kidney transplantation, Interferon, Ribavirin, Rejection

## Abstract

**Background:**

Hepatitis E virus (HEV) may be resistant to immunosuppression reduction and ribavirin treatment in kidney transplant recipients because of mutant strains and severe side effects of ribavirin which conduct to dose reduction. Sofosbuvir efficacy is controversial. Peg-interferon 2 alpha (PEG-IFN) is currently contraindicated due to a high risk of acute humoral and cellular rejection. The present study assessed, for the first time, the effect of PEG-IFN in a kidney transplant recipient infected with HEV.

**Case presentation:**

The patient had chronic active HEV that was resistant to immunosuppression reduction and optimal ribavirin treatment. He developed significant liver fibrosis. PEG-IFN was administered for 10 months, and it was well tolerated and did not induce rejection. A sustained virological response was obtained.

**Conclusions:**

We conclude that prolonged treatment with PEG-IFN in kidney transplant recipients infected with HEV could be considered as a salvage option.

## Background

Infection with hepatitis E virus (HEV) is a global public health problem that causes important morbidity and mortality, particularly in immunosuppressed patients. HEV genotype 1 and HEV genotype 2 are obligate human pathogens transmitted by the faecal-oral route via contaminated water in developing countries. In European countries, HEV genotype 3 (HEV3) and HEV genotype 4 (HEV4) are dominant and transmitted by a zoonotic route, primarily via consumption of contaminated pig meat or direct contact [1, 2].

In most European cases, HEV infection is a mild sub-clinical hepatitis and self-limiting infection that resolves spontaneously. Less than 5% [1] of patients infected with HEV3 develop symptoms of acute hepatitis, such as jaundice, elevated liver enzymes or fatigue. Progression to acute liver failure is rare, but it is more common in patients with chronic liver disease. Immunosuppressed patients may fail to clear HEV infection, which is responsible for chronic hepatitis [1–3]. HEV is also associated with extrahepatic manifestations. The most important manifestations are neurological (5.5% of patients infected by HEV3), such as Guillain-Barré syndrome, neuralgic amyotrophy, and acute meningoencephalitis [2]. Some cases of membranous and membranoproliferative glomerulonephritis were observed [1] in immunosuppressed patients infected by HEV3. HEV also induces haematological disorders, severe thrombocytopenia and aplastic anaemia.

The seroprevalence of HEV in solid organ transplant (SOT) recipients varies from 2 to 44% [2, 4]. A European study reported that the seroprevalence of HEV was roughly the same in the general population and SOT recipients and fluctuated between 7 to 17% and 8 to 18%, respectively, according to the serological test used [5]. When SOT recipients are infected with HEV, 66% will develop chronic hepatitis, which will lead to cirrhosis within 2–3 years in 14% [2, 4]. All cases of chronic HEV infection in SOT recipients were diagnosed in patients infected by HEV3 or HEV4, which supports a zoonotic mode of transmission. The sources of transmission of HEV3 in SOT patients are similar to the general population, i.e., the consumption of contaminated meat or direct contact. A few cases of HEV transmission via blood transfusions or infected graft were reported [2, 4].

**Fig. 1:**
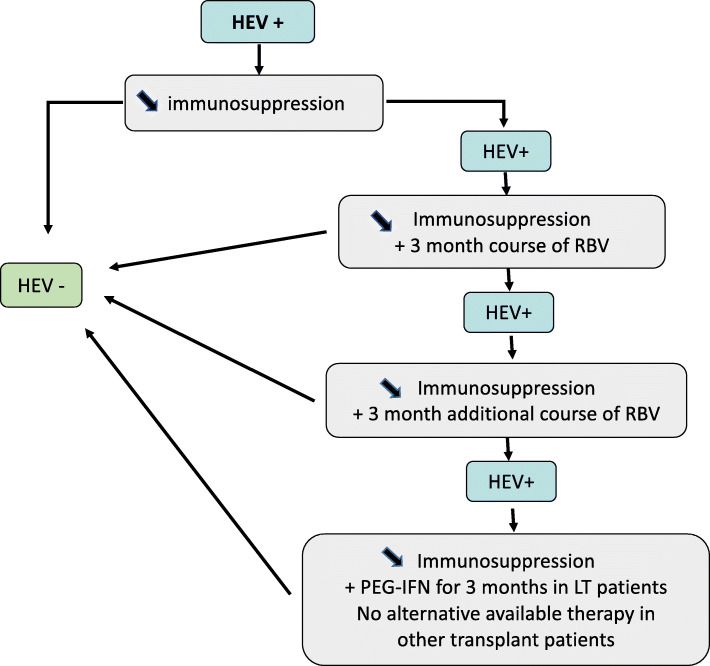
Treatment algorithm for solid organ transplant patients with chronic HEV infection (adapted from EASL Guidelines 2018)

A treatment algorithm for chronic HEV infection in immunosuppressed transplant patients, based on EASL guidelines published in 2018 [1], is presented in Fig. 1. Immunosuppression reduction generally allows a clearing of the virus in 32% of cases [1, 3, 4, 6]. When chronic HEV persists despite immunosuppression reduction, the first line treatment is at least a 3-month course of ribavirin (RBV**).** An initial median daily dose of 600 mg RBV achieved a sustained virological response (SVR) (defined as undetectable HEV RNA in the serum at least 6 months after the completion of treatment) in 78% of cases. The RBV dose varies widely between studies (400–1200 mg/d) depending on weight, haemoglobin and glomerular filtration rate adaptations [1–4, 6–8]. In patients with persisting replication in the serum and stools, an additional 3 months of treatment is proposed. In the absence of viral clearance at 6 months, Peg-interferon (PEG-IFN) may be considered only in liver transplant (LTR) patients, but not other transplant patients. In practice, the second line treatment is often an increase in RBV to 1200 mg/day for a 6–9 months [1, 3, 4], which allows an 85% SVR [1, 4]. This dose of RBV may induce anaemia, which requires erythropoietin (EPO) in 40% of patients and sometimes blood transfusions [1, 3, 4, 6, 8]. The 25% treatment failure in SOT are generally linked to RBV dose reduction due to severe side effects and mutant strains, such as HEV polymerase variant (G1634R) [1, 6, 7].

Other treatments were tested after RBV failure, such as sofosbuvir, which decreased HEV viral load, but its ability to cure HEV infection, even in combination with RBV, remains controversial [9–12]^.^ To date, a single case of SVR with sofosbuvir plus RBV was observed in a kidney transplant recipient (KTR) [10].

PEG IFN 2 alpha was used in LTR patients infected by hepatitis C (HCV) with a 50% SVR, but it was contra-indicated in other SOTs due to a higher expected risk of acute humoral and cellular rejection [4, 13–16]. Similarly, PEG-IFN was successfully prescribed in 5 LTR patients infected by HEV [3] but not in other SOTs.

We report a case of chronic HEV infection in a kidney transplant recipient treated with PEG-IFN after failure of a decrease in immunosuppression level associated with a well-performed RBV treatment. The patient did not present cellular or humoral graft rejection and was cured from HEV.

## Case presentation

A 47-year-old KTR was diagnosed with chronic genotype 3i (determination of genotype by sequencing of ORF2 region [17]) HEV infection 4.5 years after transplantation for an undetermined glomerulonephritis. At the time of diagnosis, alanine aminotransferase was 117 IU/L, HEV plasma viral load (VL) was 6 log IU/mL (Altona Diagnostics / RealStar® HEV RT-PCR kit) and liver stiffness measurement (LSM) was 10.6 kPa on transient elastography.

The beginning of the infection was at least 2 years prior because a plasma sample at that time was retrospectively found to be positive for HEV with a viral load of 6.54 log IU/mL. The patient had no extrahepatic manifestations of HEV infection. He was treated with tacrolimus (TAC) 1 mg X 2/day (d) (trough level (TL) 12.3 ng/ml) and mycophenolate mofetil (MMF) 750 mg X 2/d. TAC was reduced to 0.5 mg X 2/d (TL 4.2 ng/ml). At the same time, RBV 800 mg/d was introduced (58 kg, haemoglobin (Hb) 14.7 g/dL) but suspended after 2 weeks because of anaemia (Hb 8.0 g/dL) that required 2 units of packed red blood cells at week (W) 4 (Hb 6.8 g/dL) and EPO 10,000 IU weekly. After this short RBV course, transaminases were normalized, and HEV viremia was undetectable.

**Fig. 2:**
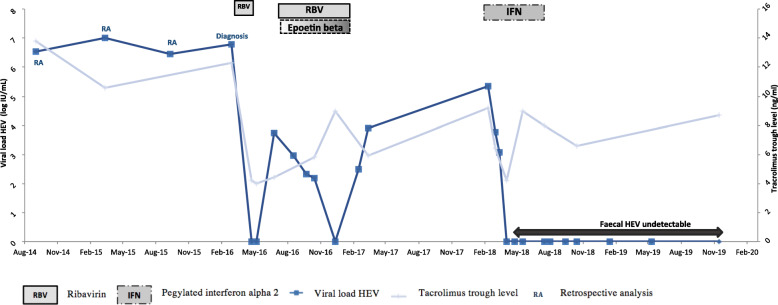
Evolution of viral load HEV and Tacrolimus

However, HEV viremia was detectable 2 months later (3.75 log IU/mL). RBV (200 mg/d) was reintroduced (W0) and increased to a maximum of 800 mg/d with many adjustments to Hb levels and an injection of EPO 30,000 IU/wk. Due to a low TL (2.25 ng/mL), TAC was increased to 1 mg × 2/d (TL 9 ng/ml). At W11, plasma VL was 2 log IU/mL. At W22, the patient received another packed red blood cell treatment for symptomatic anaemia (Hb 7.8 g/dL). EPO was increased to 30,000 IU × 2/wk. At W23, the plasma VL was undetectable. RBV at 400 mg–600 mg/d was continued with EPO 30,000 IU × 2/wk., until W31, but plasma VL became positive again (2.50 log IU/mL) and treatment was stopped. To identify mutations associated with RBV failure, sequencing of the polymerase region was made at the French National Reference Center for HEV (Pr Jacques Izopet, Toulouse) by the Sanger method [18]. The sequencing of the polymerase region revealed the presence of 3 mutations (V1479I - Y1587F - G1634G/R). Seven months after the end of RBV treatment, viremia remained positive, LSM was 12.6 kPa, and liver biopsy showed fibrosis progression (METAVIR score A1F2). PEG-IFN (90 μg/wk. subcutaneously) was introduced for 2 weeks, then 135 μg/wk., with close monitoring of serum creatinine and proteinuria. Immunosuppression was also reduced to MMF 500 mg × 2/d and TAC 0.5 mg × 2/d (TL 6.8 ng/ml) then modified for TAC-XR 0.5 mg/d due to a high TL (9.2 ng/ml). After 6 weeks of treatment, no sign of cellular rejection or donor-specific HLA antibodies (DSAs) were observed, and HEV viremia and faecal RT-PCR were negative. Immunosuppressive therapy was re-increased (TAC-XR 1 mg/day then 2 mg/day) due to a low TL TAC-XR (3.1 ng/mL), and PEG-IFN was continued 3 months after faecal RT-PCR was negative, for a total administration of 6 months. LSM at the end of treatment was 6.3 kPa, and RT-PCR remained negative in plasma and faeces. HEV remains undetectable in plasma and faeces 16 months after PEG-IFN discontinuation (Fig. 2). LSM was 5.1 kPa, and no sign of kidney rejection was observed (DSA negative).

## Discussion and conclusions

This paper is the first report of chronic HEV infection treated with PEG-IFN in a KTR. The most remarkable result was that the patient was cured of HEV without kidney rejection. The patient underwent careful surveillance. Tolerance was good compared with RBV alone. PEG-IFN was administered for approximately 10 months, and it was not associated with any sign of graft rejection during the course of treatment or after 16 months. Late rejection has been described, especially in longer lengths of therapy in chronic HCV, and an extended close monitoring was required [16]. We also administered PEG-IFN in 2 HEV-infected heart transplants without any graft rejection (unpublished personal data), but treatment was unsuccessful.

Therapeutic alternatives in cases of intolerance or immunomodulation and RBV failure in SOTs remain unknown. Our patient was already treated according to standard recommendations: immunosuppression was lowered as much as possible, and the doses and duration of RBV were optimized most of the time, with adjunctive administration of EPO and blood transfusion when necessary. The results of the use of sofosbuvir alone or in combination with daclatasvir failed to demonstrate efficiency in vivo [9]. The use of sofosbuvir in association with RBV was not conclusive. In the single case of an SVR using sofosbuvir plus RBV in a KTR, RBV was doubled from 600 mg/d to 1200 mg/d during the combined therapy, so the RBV alone may be responsible for SVR [10].

Rapid progression towards cirrhosis is frequent in immunosuppressed patients, especially in kidney and heart transplant recipients [19, 20]. PEG-IFN was used as an alternative to RBV monotherapy in liver transplant recipients. However, it was successfully prescribed in only 5 patients [3]. In contrast, because of its immunomodulatory effect, PEG-IFN was associated with a high risk of humoral and cellular rejection and renal failure in the context of HCV in kidney and liver transplant recipients, and it is contraindicated for SOT recipients other than the liver [3, 4]. Here, PEG-IFN successfully cured chronic HEV. RT-PCR was undetectable in plasma and faeces after 6 weeks. PEG-INF for chronic HEV in LTR was administered for 3 to 12 months in a previous study, and the dose varied from 135 to 180 μg/week [3]. We empirically prolonged treatment 12 weeks after virological clearance for a total of 6 months.

PEG-IFN treatment was successful despite the occurrence of 3 HEV polymerase variants, including the G1634R variant, after RBV treatment. RBV applies mutagenic pressure, which may result in an increment in quasispecies diversity or viral excretion in non-responding patients. The variants selected by RBV are characterized by mutations or single nucleotide variations (SNV). In a study of 63 SOT patients with chronic hepatitis E (genotype 3), the authors found that although its proportion was increased in patients whose RBV treatment failed, the presence of the SNV G1634R did not compromise the response to a second RBV treatment [7]. Another study highlighted the emergence of SNV G1634R during RBV treatment and subsequent treatment failure, and suggested that this mutation contributed to resistance [6].

In conclusion, in cases of RBV first line treatment failure (associated with a decrease of immunosuppression), prolonged treatment with PEG-IFN in kidney transplant recipients infected by HEV may be safe without acute or delayed graft rejection.

## Data Availability

The datasets used and/or analysed during the current study are available from the corresponding author on reasonable request.

## Abbreviations

D: Day
DSAs: Donor-specific HLA antibody
EPO: Erythropoietin
Hb: Haemoglobin
HCV: Hepatitis C Virus
HEV: Hepatitis E Virus
KTR: Kidney transplant recipient
LSM: Liver stiffness measurement
LTR: Liver transplant recipients
MMF: Mycophenolate mofetil
PEG-IFN: Peg-interferon 2 alpha
RBV: Ribavirin
SNV: Single nucleotide variations
SOT: Solid organ transplant
SVR: Sustained virological response rate
TAC: Tacrolimus
TL: Trough level
VL: Plasma viral load
W: Week
